# Synthesis, Characterization, and Optimization of Magnetoelectric BaTiO_3_–Iron Oxide Core–Shell Nanoparticles

**DOI:** 10.3390/nano10030563

**Published:** 2020-03-20

**Authors:** Mahmud Reaz, Ariful Haque, Kartik Ghosh

**Affiliations:** 1 Department of Physics, Astronomy and Materials Science, Missouri State University, Springfield, MO 65897, USA; mahmud.reaz@vanderbilt.edu (M.R.); KartikGhosh@MissouriState.edu (K.G.); 2 Interdisciplinary Materials Science, Vanderbilt University, Nashville, TN 37235, USA; 3 Department of Materials Science and Engineering, North Carolina State University, Raleigh, NC 27695, USA

**Keywords:** nanoparticles, oxide-nanomaterials synthesis, core–shell, perovskite oxide, superparamagnetism, magnetic iron-oxides, energy-dispersive X-ray spectroscopy

## Abstract

Improvement of magnetic, electronic, optical, and catalytic properties in cutting-edge technologies including drug delivery, energy storage, magnetic transistor, and spintronics requires novel nanomaterials. This article discusses the unique, clean, and homogeneous physiochemical synthesis of BaTiO_3_/iron oxide core–shell nanoparticles with interfaces between ferroelectric and ferromagnetic materials. High-resolution transmission electron microscopy displayed the distinguished disparity between the core and shell of the synthesized nanoparticles. Elemental mapping and line scan confirmed the formation of the core–shell structure. Energy-dispersive X-ray spectroscopy and X-ray photoelectron spectroscopy detected the surface iron oxide phase as maghemite. Rietveld analysis of the X-ray diffraction data labeled the crystallinity and phase purity. This study provides a promising platform for the desirable property development of the futuristic multifunctional nanodevices.

## 1. Introduction

Nanomaterials with both ferromagnetic and ferroelectric properties are useful for compact devices and sensors [1,2,3]. Coupling between electric and magnetic properties (e.g., magnetic polarization from electric field and vice versa) is essential for several technologies. Application of the magnetoelectric (ME) effect-based devices would include but not limited to micro- and nanoelectromechanical systems (MEMS and NEMS), data storage media, spintronics, spin valves, nonvolatile random access memories, and so on [4,5,6]. Unlike compound multiferroic materials, which is a mixture of materials without an interface, the composite structure with ferroelectric and magnetic interfaces typically yields an excellent ME coupling response. ME coupling is a known effect in thin films, heterostructures, ferrite composites, transition metals, alloys, and core–shell nanoparticles (CSNP) [7]. However, the core–shell structure with spherical interfaces that connect ferroelectric and antiferromagnetic materials in atomic dimensions would be an ideal candidate for next-generation multiferroics. As coupling originates from the strain transfer at the boundary, the large surface area-to-volume ratio in nanomaterials enhances ME coupling. Theoretical studies also support stronger coupling in core–shell type nanostructures of spherical, rectangular, and cylindrical shapes [8]. When a functional material exists in more than one crystal structure, it is easier to tune the properties by polymorphic transition. Polymorphism is a molecular level change in the lattices and occurs when the field strength from atomic interaction surpasses the energy of the noninteracting part of the system. Both barium titanate (BaTiO_3_) and iron oxide are exceptional multifunctional materials for two reasons, where the second reason is more important than the first. First, both of them are stable in more than one molecular phase without losing their signature properties. Second, both are technologically important for the exceptional ferroelectric and ferromagnetic response, respectively. Ferroelectric BaTiO_3_ is a well-studied perovskite-structured material with high permittivity. Barium titanate exhibits successive phase transitions from orthorhombic to rhombohedral, tetragonal to orthorhombic, and cubic to tetragonal at 183, 278, and 393 K, respectively [9,10]. All four phases of barium titanate are either ferro- or antiferromagnetic [11]. On the other hand, the maghemite phase of iron oxide with an inverse spinel ferrite structure shows ferrimagnetic ordering up to a very high temperature (Néel temperature ~950 K). High chemical stability, room temperature conductivity, and biocompatibility make maghemite suitable for industrial processes. In addition, magnetic moments in maghemite are tunable with substitutional atoms or dopants. Thus, iron oxide has become a material of interest for application in spintronics, drug delivery, and electronic recording media [12,13,14]. Combining the properties of superparamagnetic maghemite with ferroelectric barium titanate, the goal of this study was to synthesize ferroelectric–superparamagnetic composite nanostructures with extended interfaces.

In CSNP, property modification via postprocessing is easier for surface material. Unlike core, shell surfaces remain active and interact easily with the ambient. Property modulation of magnetic oxides in previous studies via surface treatment [15,16] (annealing/reduction/oxidation in reducing ambient) justified our choice of coating barium titanate with iron oxide. Previously, researchers synthesized Fe_2_O_3_–BaTiO_3_ CSNP with Fe_2_O_3_ as the core and BaTiO_3_ as the shell component [17,18], which was useful but did not offer similar versatility. Another group investigated the desirable BaTiO_3_–Fe_2_O_3_ structures; but, they ended up getting at least 16 different quaternary compounds [19,20]. Mornet et al. have claimed to synthesize BaTiO_3_-Fe_2_O_3_ CSNP; however, their results lacked evidence from structural, elemental, and surface characterization techniques [21]. Moreover, the grain size of the nanoparticles of their CSNP was 250 nm and was not truly relevant for the cutting-edge nanodevices, as the surface area-to-volume ratio was much lower.

In this study, we presented a controlled synthesis method to obtain multiferroic BaTiO_3_–γFe_2_O_3_ core–shell nanostructures for the first time, with the aim to achieve a ME coupling at the interface of nanocomposites. Following a series of sonication and centrifugation processes, subsequent annealing in the oxygen environment provided a clean synthesis of perovskite oxide–iron oxide CSNP. Synthesized multiferroic nanoparticles (NP) showed excellent uniformity in size and shape. This novel process of chemical synthesis with distinct crystal structures may serve as a potential template to prepare other nanostructures, especially with oxide materials. The CSNP were characterized by scanning electron microscopy, X-ray diffraction, and X-ray photoelectron spectroscopy that conclusively showed the formation of homogeneous core–shell nanostructures instead of particles with distinct phases. We also reported a detailed systematic investigation on magnetic properties of BaTiO_3_–γFe_2_O_3_ CSNP with controlled shell thickness. The obtained structural property results implied that electric/magnetic core-shell nanoparticles would be useful for tunable ME devices.

## 2. Experimental Methods

### 2.1. CSNP Synthesis

**Figure 1:**
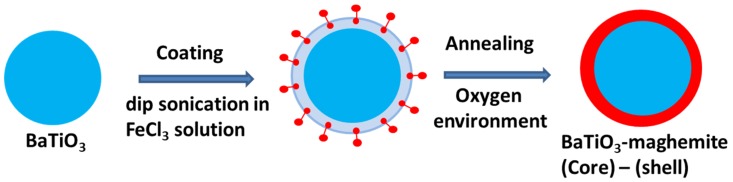
Schematic illustration of the synthesis of perovskite oxide/ferrite core–shell nanostructures.

Thin iron oxide coating on BaTiO_3_ nanospheres was developed in two steps, as illustrated in Figure 1. Both primary materials (namely, FeCl_3_·6H_2_O and BaTiO_3_) used in the synthesis were commercially purchased. In an aqueous solution, iron chloride dissociates into ionic constituents, but barium titanate is insoluble. A series of sonication and centrifugation processes coated the BaTiO_3_ NPs with the iron and chloride ions. Initial sonication dispersed the nanoparticles for uniform coating via physisorption. Subsequent centrifugation, which is a density-based separation method in fluids, separated nanoparticles from excess iron and chlorine ions via precipitation. After each centrifugation, precipitates were mixed with fresh double-deionized waterwater. Intermittent sonication helped mobilize the access ions that were either weakly or not bonded with CSNP. Our primary sample (s1) was synthesized from an initial 1:2 weight ratio of BaTiO_3_ NPs and FeCl_3_·6H_2_O. Annealing for 2 h in oxygen ambient at 500 °C oxidized chlorine, and the surface phase became iron oxide.

### 2.2. XPS

X-ray photoelectron spectroscopy (XPS) is a widely accepted characterization technique for probing CSNP surface/interface. A Thermo Scientific Alpha 110 hemispherical analyzer (Waltham, MA, USA) with a pass energy of 25 eV was used for the measurements. X-ray from an Al-K_α_ source (1486.6 eV) characterized the chemical environment of the surface atoms. Flood gun compensated the surface charge during data acquisition. The lower and upper bound of the kinetic energy for this XPS system were 100 and 1300 eV, respectively. Aperture value remained constant throughout the measurements. The CasaXPS 2.3.16 software (Devon, UK) was used for the analysis and peak fitting of the XPS spectra. The system was calibrated with respect to the carbon 1s peak (284.8 eV). A Shirley model within the CasaXPS software simulated the background noise to fit data. A Gaussian–Lorentzian product-based function (GL-30) was used for fitting symmetrical line shapes, whereas a Gaussian-Lorentzian convoluted function (LA-a,b,n) fitted asymmetric line shapes of the high-resolution peaks.

### 2.3. TEM

Transmission electron microscope (TEM) images of the BaTiO_3_/iron oxide CSNP were acquired using a FEI Talos microscope (Waltham, MA, USA). Energy-dispersive X-ray analysis (EDS) using a high-angle annular dark-field (HAADF) scanning TEM decomposed the elemental configuration. The electron field emission source of the TEM was operated at 200 keV. The plasma etching of CSNP removed any unwanted organic and inorganic residue from the sample surface before the TEM analysis. Line scan in the oxide–perovskite interface verified the core–shell nature of the NPs.

### 2.4. XRD

Room temperature X-ray diffraction (XRD) measurements (θ–2θ scan) conducted with a powder diffractometer (Bruker D8 Discover sourced from Billerica, MA, USA) probed the structural composition of CNSPs. The XRD system included a Cu k_α_ radiation source and a state of the art LYNXEYE XE detector. X-ray source was operated with a 40 kV voltage and 25 mA current in an ultrahigh vacuum chamber. LYNXEYE detector filtered fluorescence and K_β_ radiation. Secondary monochromators and metal filters minimized the intensity loss and noise, especially near absorption edge energies. The full-pattern refinement program using TOPAS software from Bruker (Billerica, MA, USA), calculated the structural parameters by comparing XRD data with the crystallographic models. A modified ThompsonCox-Hastings pseudo-Voigt peak function (TCHZ) handled the zero error and detected the incident beam profile during refinement. The TCHZ was in line with the NIST 674b standard reference library. Calibrated parameters in TCHZ reflected the characteristics and axial divergence of the incident beam profile. Common TCHZ parameters were used for all XRD data. Background noise was subtracted from the XRD pattern using a Chebyshev Polynomial of 5th order. A nonlinear least square regression minimized the value of “R-weighted pattern” (Rwp) and facilitated a better convergence in Rietveld refinement.

### 2.5. Magnetic Measurements

The field vs. magnetization measurements were performed by a superconducting quantum interference device (SQUID) magnetometer (Quantum Design, MPMS 5XL, California, CA, USA). Under a large variation of the magnetic field (−10,000 to +10,000 Oe), the magnetometer measures the hysteresis (M–H curve). The sensitivity of the magnetometer was 10^−9^ emu. All the data were analyzed using the Origin Pro 8.5.1 software.

## 3. Results and Discussion

**Figure 2:**
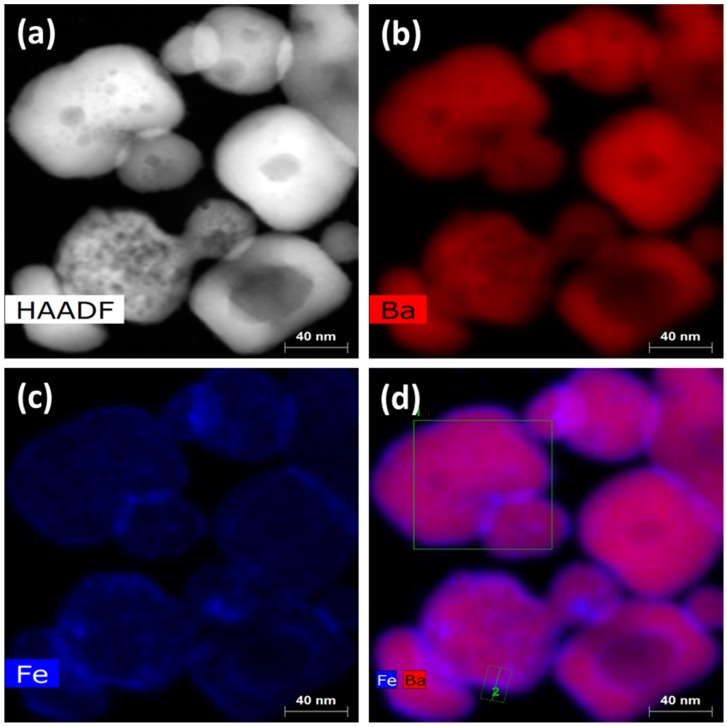
Elemental distributions in core–shell nanoparticles (CSNP) via TEM color map show (**a**) sample area for high-angle annular dark-field (HAADF) imaging. Elemental mapping of (**b**) only Ba atoms, (**c**) only Fe atoms, and (**d**) both Fe and Ba atoms.

TEM imaging illustrated the overall topography and distribution of the atoms in the core and at the surface. TEM images in Figure 2 indicate an acceptable dispersion of the CSNP. The HAADF image in Figure 2a shows the sample area used for elemental mapping. Each nanoparticle in Figure 2b shows a uniform density of barium. Figure 2c shows an increasing number of iron atoms (intense color) near the surfaces/interfaces. Iron atoms in Figure 2d encompass the barium titanate and clearly established the core–shell nature of the synthesized NPs. However, in spherical symmetry, higher Fe count near the NP boundary required an additional discussion. For a directional beam, the highest number of interactions with spherical surfaces occurred in a vertical cross section near the perimeter of the NPs as shown in Figure 2d. Here, the captured interaction is 3D in nature, but the resultant data are two dimensional.

**Figure 3:**
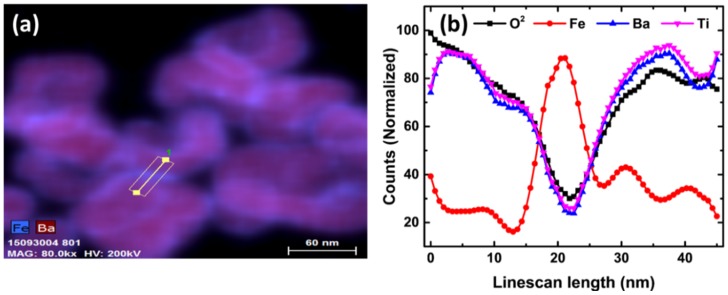
(**a**) TEM-EDX showing Fe and Ba intensities at the region of interest for the quantitative analysis (yellow line) and (**b**) normalized intensity along the yellow line.

The line scan of the CSNP as shown in Figure 3 complements our claim on successful core–shell formation. Line scan across the interfaces (yellow straight line) in Figure 3a that is plotted in Figure 3b provides conclusive evidence of the incremental Fe count adjacent to the interface. In the interface, barium and titanium count dipped. In contrast, slightly higher oxygen count indicated the possibility of the core–shell formation (iron oxide over BaTiO_3_ NPs). Far from the interfaces, both Ba and Ti count increased, and ruled out the presence of any homo- or heterogeneous mixture of constituents.

**Figure 4:**
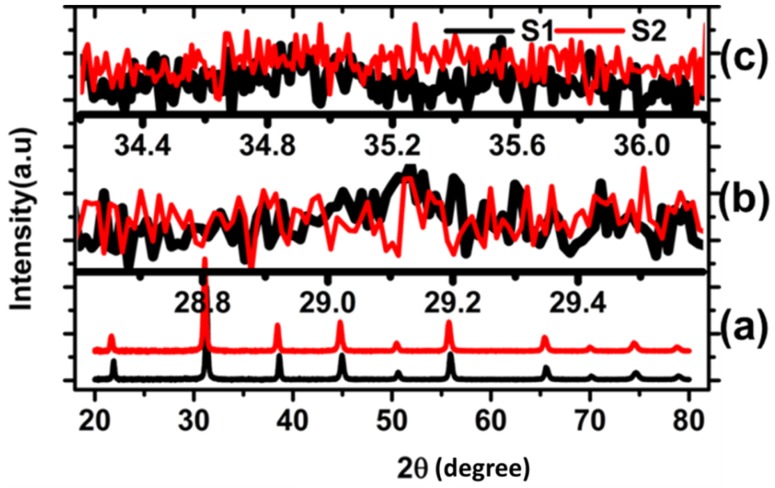
XRD diffraction data of oxidized CSNP that were synthesized from low (s1) and high (s2) initial concentration of FeCl_3_·6H_2_O. (**a**) Bragg peak from crystalline BaTiO_3_, references were shifted in vertical scale, (**b**) zoomed in near (220) peak of maghemite, and (**c**) zoomed in near (311) peak of maghemite.

The XRD diffraction patterns in Figure 4a probe the crystallinity features of CSNP. No impurity phases were detected in the diffraction pattern signifying the high quality of the synthesis method. To show that the core–shell formulation was independent of increasing FeCl_3_·6H_2_O concentration in the primary solution, we included two samples (synthesized using high and low FeCl_3_·6H_2_O concentrations), namely, $s_{1}$ and $s_{2}$. Both samples showed similar phase percentages when fitted with BaTiO_3_ and ϒ-Fe_2_O_3_ phases. As the initial weight ratio of $s_{1}$ and $s_{2}$ showed no variation on their XRD patterns, sample $s_{2}$ was not considered for further characterization presented in the later sections.

Rietveld structure refinement using TOPAS calculated a crystal size close to 60 nm, which is an acceptable deviation from the commercial spec (50 nm). Successive diffraction peaks from low to high Bragg angle, as shown in the Figure 4a, corresponds to the (100), (110), (111), (002), (200), (201), (210), (211), (220), (221), (301), and (311) planes of BaTiO_3_, and were in agreement with the Rietveld refinement that uses crystallographic information file from Inorganic Crystal Structure Database. The ordered crystalline structure of iron oxide was not clearly identified in Figure 4a.

If the maghemite phase exists within CSNP, two major diffraction peaks are expected at 29.15° and 35.2°, respectively. Diffraction patterns near 29.15° in Figure 4b suggest a possible presence of maghemite, with a broad low-intensity peak for (220) planes near 29.15°. A similar peak for another plane, such as (311), could not be detected as illustrated near 35.2° in Figure 4c. Only a handful of atomic planes in the shell nanolayer limited us to go beyond the observed ultralow intensity and high full width at half maximum (FWHM)diffraction data that we believed were coming from the maghemite. LYNXEYE XE detector used to acquire XRD was widely regarded as the state of the art of our time and offered an advantage with intensity, peak-to-background ratio, lower limits of detection, and accurate profile fitting over other conventional techniques.

**Figure 5:**
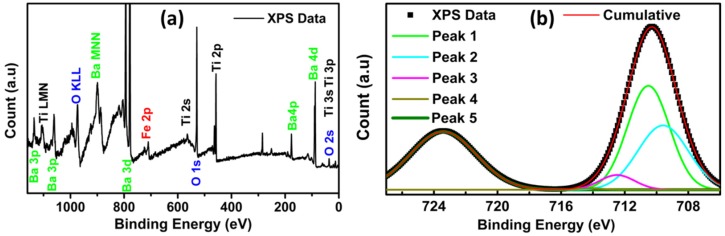
(**a**) Background-subtracted XPS scan of the BaTiO_3_/iron oxide CSNP. (**b**) Sherley background-subtracted high-resolution scan data of the Fe 2p^3/2^ orbital deconvoluted without surface peaks.

Surface-sensitive XPS technique showed a significantly higher quantity of iron (3.05%) in comparison to a bulk method like EDX (0.47%) and corroborated successful CSNP synthesis. In addition, quantitatively a low atomic count of the Ba and Ti from the XPS (surface sensitive) further supported the core–shell nature of the synthesized nanostructures. Figure 5a shows the background-subtracted XPS scan of the BaTiO_3_/iron oxide CSNP. Relevant Fe, Ba, Ti, and O peaks in the CSNP structure were identified. Similar color labeled the XPS and Auger peaks from an individual element. The primary XPS scan recognized the presence of the iron and further decomposed around binding energies as shown in Figure 5b.

To obtain the true surface information of the CSNP, we investigated the high-resolution scan of Fe 2p orbital in Figure 5b. No shoulder peak around Fe 2p^3/2^ binding energies ruled out the presence of any metallic iron phase in the sample. We also did not observe any satellites from Fe^3+^ or Fe^2+^ state in the scan, which signified that iron was not present in multiple phases. Around 13 eV, the binding energy difference between the 3/2 and 1/2 orbital suggested the stoichiometric consistency of iron and oxygen. Within a few nanometer length scale, maghemite (Fe^3+^ dominant stoichiometry, ϒ-Fe_2_O_3_) was reported to be the most thermodynamically stable [22]. Since XRD results also indicated the presence of this inverse spinal phase, XPS Fe 2p^3/2^ peak was fitted with maghemite deconvoluted peaks from the literature [23,24]. The binding energy difference of 2.89 eV between peaks 2 and 3 agreed well with the maghemite stoichiometry. Small FWHM of peaks (1–4) supported the presence of ϒ-Fe_2_O_3_ phase in CSNP structures. All peaks were deconvoluted using the Gaussian parameters. Surface and satellite peaks were not considered during the deconvolution of the Fe 2p^3/2^ orbital. We used the Origin 8.5 Pro for fitting the Fe 2p^3/2^ peaks. Shirley backgrounds were subtracted using the CASA software. The energy, area, and FWHM of the fitted curves are summarized in Table 1.

## 4. Magnetic Measurements

**Figure 6:**
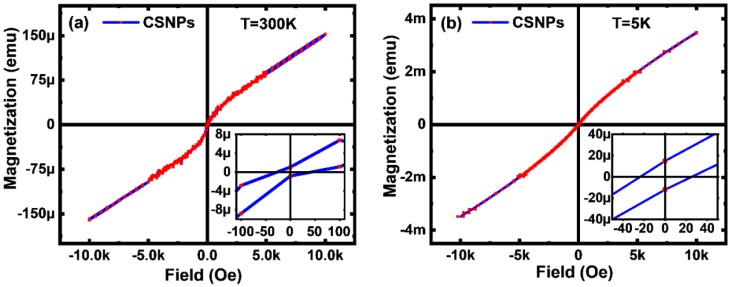
Field dependence of magnetization for the CSNP at (**a**) 300 K and (**b**) 5 K. Inset in the figure shows the low field fragment of the hysteresis loop. Scaling of the vertical axis is different (micro vs. milli) in (**a**) and (**b**).

The field dependence of magnetization at 300 K in Figure 6a is in agreement with the previous reports by other groups [25,26]. A tiny remnant magnetization and coercive field at room temperature clearly showed the superparamagnetic behavior [27,28]. In a superparamagnetic material, magnetization beyond Neel relaxation time is zero on average, and susceptibility is much higher than the paramagnets. Figure 6 agrees well with the known tendency of the single domain NPs to become superparamagnetic [29]. At close to liquid helium temperature, as shown in Figure 6b, residual magnetization increased five times, but the coercive enhancement only doubled. Such negligible coercivity at both 5 K and room temperature established the CSNP as an stable superparamagnetic material [30]. It is possible to further modify the superparamagnetic response using a surfactant coating [31]. Unidirectional anisotropy or loop shift in the hysteresis curves were small since there is no ferromagnetic–antiferromagnetic exchange bias in CSNP.

## 5. Implication of the Results

In CSNP, individual properties of the constituents get coupled via structural modification at the atomic level and subsequent characterization revealed their modified behavior. Nanospheres, including a barium titanate core and iron oxide shell, combined ferroelectric and ferrimagnetic properties via a coupling. Controlled synthesis extracted the optimal property from these multimaterial surface/interfaces. Although ME effect was not measured for our samples, previous studies strongly suggested that an electric field was able to alter the magnetism in barium titanate–iron oxide composites [32]. Considering a defined interface and spherical symmetry in our synthesized NPs, we expected a significant enhancement of the electric field-induced magnetism control, which is the key for improved device performance.

Iron oxides in the surface of CSNP are well suited for structural modification to achieve desired ferromagnetic, ferromagnetic, and antiferromagnetic properties. Iron oxide phases [33] such as hematite [α-Fe_2_O_3_], magnetite [Fe_3_O_4_], and maghemite [γ-Fe_2_O_3_] are widely known for distinct magnetic response owing to polymorphism, which causes temperature-induced phase transition. Previous studies on cobalt oxides (0.5–3 nm thick), which are transition metal oxides, magnetic, and structurally similar to iron oxides, showed annealing reduction-mediated modulation in magnetic field [34]. Changes in the magnetic property for cobalt oxides were achieved by the structural phase transition, which occurred under reduction annealing in high vacuum. A similar phase transition was achieved for iron oxide NPs under high vacuum annealing [35]. Accordingly, maghemite in the outer layer of our synthesized CSNP can be converted into other useful iron oxide phases for multiferroics applications. Controlled annealing of the CSNP by oxidation or reduction in different environments would be a reasonable step to achieve structure–property modifications. Altering key parameters (e.g., temperature, pressure, and size) during the oxidation/reduction process tuned the CSNP to meet the application-oriented specs. Uniformity in the coating and dispersion of the CSNP in this work was advantageous for the multiferroic applications.

Our approach combining nanotechnology and solid-state chemistry methods opened the way to the building of innovative materials for integration and multifunctionality. Specifically, these novel multiferroic CSNP have exciting applications, such as magnetic tweezers, protein and DNA separators, therapeutic agents for hyperthermia, MRI contrast agent, and radioactive isotopes for radio- and chemotherapy.

## 6. Summary

We have successfully synthesized superior-quality BaTiO_3_/iron oxide CSNP by the physiochemical synthesis process, and characterized those nanoparticles to show the key structural difference between the core and the shell regions. The bulk (XRD) and surface (XPS) measurements support our claim on the clean synthesis of the multifunctional CSNP. The TEM analysis conclusively shows the regions of two different phases in the nanoparticles at the core and at the outer shell regions, which rules out the possibility of the presence of any single-phase particles. Detail XPS analysis in this study may serve as a framework to analyze similar iron oxide surface structures. The observed superparamagnetic response due to the thin nanolayer of iron oxide has a wide variety of device applications. Our two-step synthesis method may be applicable for other core–shell-type systems, especially with oxide materials.

## Figures and Tables

**Table 1:** Deconvoluted Gaussian peak parameters of peaks 1–5 from Figure 5b.

Name	Position (eV)	Area	FWHM
Peak1	710.5126	352.9095	3.12362
Peak2	709.5909	260.5075	3.72701
Peak3	712.4837	41.63253	2.6034
Peak4	714.143	2.90822	2.90822
Peak5	723.4224	310.1352	4.91504

## References

[1] Mamun M.A.-A., Haque A., Pelton A., Paul B., Ghosh K. (2018). Structural, Electronic, and Magnetic Analysis and Device Characterization of Ferroelectric-Ferromagnetic Heterostructure (BZT-BCT/LSMO/LAO) Devices for Multiferroic Applications. IEEE Trans. Magn..

[2] Vopson M.M. (2015). Fundamentals of Multiferroic Materials and Their Possible Applications. Crit. Rev. Solid State Mater. Sci..

[3] Haque A., Mahbub A.R., Abdullah-Al Mamun M., Reaz M., Ghosh K. (2019). Fabrication and thickness-dependent magnetic studies of tunable multiferroic heterostructures (CFO/LSMO/LAO). Appl. Phys. A.

[4] Zhao S. (2015). Advances in Multiferroic Nanomaterials Assembled with Clusters. J Nanomater..

[5] Mamun M.A.-A., Haque A., Pelton A., Paul B., Ghosh K. (2019). Fabrication and ferromagnetic resonance study of BZT-BCT/LSMO heterostructure films on LAO and Pt. J. Magn. Magn. Mater..

[6] Ali T., Gigli L., Ali A., Khan M.N. (2019). Structural transformation and inverse magnetocaloric effect in Ni_50_Mn_33_In_17_. J. Magn. Magn. Mater..

[7] Sreenivasulu G., Popov M., Chavez F.A., Hamilton S.L., Lehto P.R., Srinivasan G. (2014). Controlled self-assembly of multiferroic core-shell nanoparticles exhibiting strong magneto-electric effects. Appl. Phys. Lett..

[8] Kukhar V.G., Pertsev N.A., Kholkin A.L. (2010). Thermodynamic theory of strain-mediated direct magnetoelectric effect in multiferroic film–substrate hybrids. Nanotechnology.

[9] Kobori H., Uzimoto K., Hoshino A., Yamasaki A., Sugimura A., Taniguchi T., Horie T., Naitoh Y., Shimizu T. (2012). Magnetoresistance Intensification of Fe_3_O_4_/BaTiO_3_ Nanoparticle-Composite-Sinter Produced by Low Temperature Heat Treatment. J. Supercond. Nov. Magn..

[10] Singh S., Kumar N., Jha A., Sahni M., Sung K., Jung J.H., Chaubey S. (2015). Study of magnetic, dielectric and magnetodielectric properties of BaTiO_3_/Fe_3_O_4_ core/shell nanocomposite. J. Mater. Sci. Mater. Electron..

[11] Zhang Q., Cagin T., Goddard W.A. (2006). The ferroelectric and cubic phases in BaTiO_3_ ferroelectrics are also antiferroelectric. Proc. Natl. Acad. Sci. USA.

[12] Pankhurst Q.A., Connolly J., Jones S.K., Dobson J. (2003). Applications of magnetic nanoparticles in biomedicine. J. Phys. Appl. Phys..

[13] Dronskowski R. (2001). The Little Maghemite Story: A Classic Functional Material. Adv. Funct. Mater..

[14] Haque A., Sumaiya S. (2017). An Overview on the Formation and Processing of Nitrogen-Vacancy Photonic Centers in Diamond by Ion Implantation. J. Manuf. Mater. Process..

[15] Antón R.L., González J.A., Andrés J.P., Canales-Vázquez J., Toro J.A.D., Riveiro J.M. (2014). High-vacuum annealing reduction of Co/CoO nanoparticles. Nanotechnology.

[16] Kumar C.G., Poornachandra Y., Chandrasekhar C. (2015). Green synthesis of bacterial mediated anti-proliferative gold nanoparticles: Inducing mitotic arrest (G2/M phase) and apoptosis (intrinsic pathway). Nanoscale.

[17] Buscaglia M.T., Buscaglia V., Curecheriu L., Postolache P., Mitoseriu L., Ianculescu A.C., Vasile B.S., Zhe Z., Nanni P. (2010). Fe_2_O_3_@BaTiO_3_ Core−Shell Particles as Reactive Precursors for the Preparation of Multifunctional Composites Containing Different Magnetic Phases. Chem. Mater..

[18] Curecheriu L., Postolache P., Buscaglia M.T., Buscaglia V., Ianculescu A., Mitoseriu L. (2014). Novel magnetoelectric ceramic composites by control of the interface reactions in Fe_2_O_3_@BaTiO_3_ core-shell structures. J. Appl. Phys..

[19] Vanderah T.A., Loezos J.M., Roth R.S. (1996). Magnetic Dielectric Oxides: Subsolidus Phase Relations in the BaO:Fe_2_O_3_:TiO_2_ System. J. Solid State Chem..

[20] Siegrist T., Vanderah T.A. (2003). Combining Magnets and Dielectrics: Crystal Chemistry in the BaO−Fe_2_O_3_−TiO_2_ System. Eur. J. Inorg. Chem..

[21] Mornet S., Elissalde C., Bidault O., Weill F., Sellier E., Nguyen O., Maglione M. (2007). Ferroelectric-Based Nanocomposites: Toward Multifunctional Materials. Chem. Mater..

[22] Birkner N., Navrotsky A. (2017). Thermodynamics of manganese oxides: Sodium, potassium, and calcium birnessite and cryptomelane. Proc. Natl. Acad. Sci. USA.

[23] Grosvenor A.P., Kobe B.A., Biesinger M.C., McIntyre N.S. (2004). Investigation of multiplet splitting of Fe 2p XPS spectra and bonding in iron compounds. Surf. Interface Anal..

[24] Haque A., Mamun M.A.-A., Taufique M.F.N., Karnati P., Ghosh K. (2018). Temperature Dependent Electrical Transport Properties of High Carrier Mobility Reduced Graphene Oxide Thin Film Devices. IEEE Trans. Semicond. Manuf..

[25] Nakata K., Hu Y., Uzun O., Bakr O., Stellacci F. (2008). Chains of Superparamagnetic Nanoparticles. Adv. Mater..

[26] Li Z., Sun Q., Gao M. (2005). Preparation of Water-Soluble Magnetite Nanocrystals from Hydrated Ferric Salts in 2-Pyrrolidone: Mechanism Leading to Fe_3_O_4_. Angew. Chem. Int. Ed..

[27] Yu J., Yu X., Huang B., Zhang X., Dai Y. (2009). Hydrothermal Synthesis and Visible-light Photocatalytic Activity of Novel Cage-like Ferric Oxide Hollow Spheres. Cryst. Growth Des..

[28] Mitov I., Cherkezova-Zheleva Z., Mitrov V. (1997). Comparative Study of the Mechanochemical Activation of Magnetite (Fe_3_O_4_) and Maghemite (γ-Fe_2_O_3_). Phys. Status Solidi A.

[29] Akbarzadeh A., Samiei M., Davaran S. (2012). Magnetic nanoparticles: Preparation, physical properties, and applications in biomedicine. Nanoscale Res. Lett..

[30] Dormann J.L., Tronc E., Fiorani D. (1997). Advances in Chemical Physics.

[31] Layek S., Pandey A., Pandey A., Verma H.C. (2010). Synthesis of γ–Fe_2_O_3_ nanoparticles with crystallographic and magnetic texture. Int. J. Eng. Sci. Technol..

[32] Wang L.-M., Petracic O., Mattauch S., Koutsioumbas A., Wei X.-K., Heggen M., Leffler V., Ehlert S., Brückel T. (2018). Magnetoelectric coupling in iron oxide nanoparticle—Barium titanate composites. J. Phys. Appl. Phys..

[33] Cornell R.M., Schwertmann U. (2003). The Iron Oxides: Structure, Properties, Reactions, Occurrences and Uses.

[34] Reaz M., Haque A., Cornelison D.M., Wanekaya A., Delong R., Ghosh K. (2020). Magneto-luminescent Zinc/Iron oxide core-shell nanoparticles with tunable magnetic properties. Phys. E Low-Dimens. Syst. Nanostructures.

[35] Anupama A.V., Keune W., Sahoo B. (2017). Thermally induced phase transformation in multi-phase iron oxide nanoparticles on vacuum annealing. J. Magn. Magn. Mater..

